# Supplementary material for: Face and context integration in emotion inference is limited and variable across categories and individuals
Source: Nat Commun. 2024 Mar 19;15:2443. doi: 10.1038/s41467-024-46670-5 (PMC10948792; doi:10.1038/s41467-024-46670-5)
Supplement: Supplementary file 1 — Supplementary Information [file 41467_2024_46670_MOESM1_ESM.pdf]

Supplementary information for

Face and context integration in emotion inference is limited and variable across categories and individuals

Srishti Goel[1*], Julian Jara-Ettinger[1,2], Desmond C. Ong[3], Maria Gendron[1*]

[1]Department of Psychology, Yale University, 100 College St, New Haven, Connecticut 06520, USA.
[2]Wu Tsai Institute, Yale University, 100 College St, New Haven, Connecticut 06520, USA.
[3]Department of Psychology, The University of Texas at Austin, 108 E Dean Keeton St, Austin, Texas 78712, USA.

*Corresponding author. Email: srishti.goel@yale.edu and maria.gendron@yale.edu

Comparing rating distributions

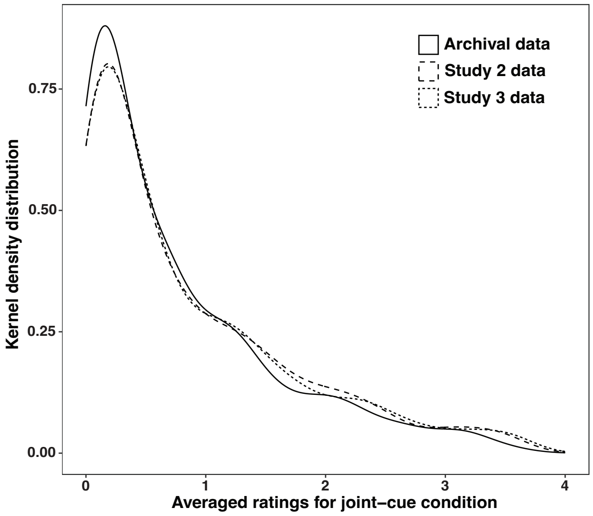

Figure S1 | Comparing joint-cue rating distribution. Smoothed density distribution of joint-cue ratings averaged across emotion for $n = 572$ observations for the common set of 44 stimuli used in the archival dataset (part of Study 1), Study 2, and Study 3. There is statistically significant high correlation of joint-cue ratings for each stimulus from archival data with Study 2 (r (570) = 0.955, $t = 76.446$, $p < 0.001$, 95% CI: 0.947, 0.961) and Study 3 (r (570) = 0.966, $t = 88.838$, $p < 0.001$, 95% CI = 0.960, 0.971) data computed using two-tailed Pearson correlation.

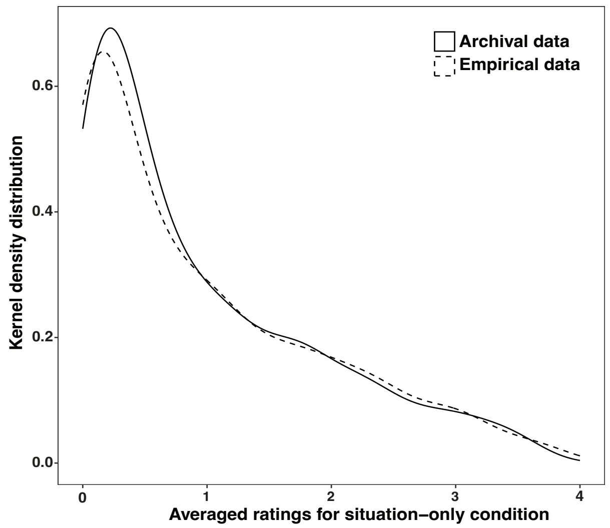

Figure S2 | Comparing situation-only rating distribution. Smoothed density distribution of situation-only ratings averaged across emotion for $n = 572$ observations from the common set of 44 stimuli. The rating distributions were obtained from archival data (part of Study 1) and an *empirical data* set that was additionally collected to validate that the samples collected for the current manuscript are comparable to the archival data. There is statistically significant high correlation between situation-only ratings for each stimulus from archival and empirical data (r (570) = 0.966, $t = 89.379$, $p < 0.001$, 95% CI: 0.960, 0.971) computed using two-tailed Pearson correlation.

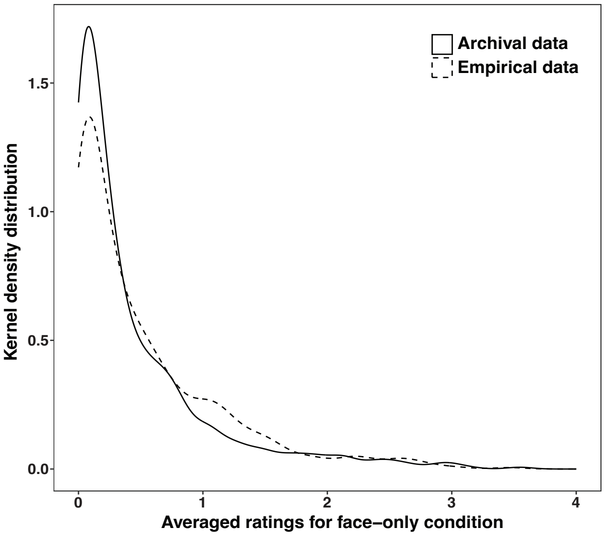

Figure S3 | Comparing face-only rating distribution. Smoothed density distribution of face-only ratings averaged across emotion for $n = 572$ observations from the common set of 44 stimuli. The rating distributions were obtained from archival data (part of Study 1) and an *empirical data* set that was additionally collected to validate that the samples collected for the current manuscript are comparable to the archival data. There is statistically significant high correlation between face-only ratings for each stimulus from archival and empirical data (r (570) = 0.934, $t = 62.458$, $p <$ 0.001, 95% CI = 0.923, 0.944) computed using two-tailed Pearson correlation.

We also performed a more conservative test of distribution comparability using the two-tailed Kolmogorov-Smirnov test. The distribution of joint-cue ratings obtained from archival data were not statistically significantly different from those obtained in Studies 2 ($D = 0.058$, $p = 0.297$) and 3 ($D = 0.052$, $p = 0.411$). The distribution of situation-only and face-only ratings obtained from archival data were significantly different from the empirical data (Situation-only: $D = 0.084$, $p =$ 0.036, Face-only: $D = 0.087$, $p = 0.025$). Visual inspection of the distributions suggests that this statistical difference is driven by the zero inflation of situation-only and face-only ratings in the archival data compared to the empirical data. However, the distribution of ratings along the intensity scale (1 to 4) is largely similar. Additionally, the high positive correlation between these rating distributions indicate that the two samples are sufficiently comparable for constructing model estimates. Indeed, model estimates for Study 2 and Study 3 computed using the *empirical* face-only and situation-only ratings (Table S1) follow the same pattern of results as observed with archival ratings (Study 1, Table S2).

Table S1. *Two-tailed Pearson correlation of Bayes cue-integration, Situation-only and Face-only model in Study 2 and 3 with bootstrapped 95% confidence intervals reported in the bracket. Here, the model estimates were computed using face-only and situation-only ratings collected from a sample of Prolific participants (empirical data) comparable to the archival sample.*

Study	Bayes cue-integration model	Situation-only model	Face-only model
Study 2	r (570) = 0.830<br>[0.799, 0.858]<br>t = 35.502<br>p < 0.001	r (570) = 0.906<br>[0.885, 0.923]<br>t = 51.157<br>p < 0.001	r (570) = 0.586<br>[0.519, 0.649]<br>t = 17.263<br>p < 0.001
Study 3	r (570) = 0.844<br>[0.815, 0.871]<br>t = 37.602<br>p < 0.001	r (570) = 0.919<br>[0.901, 0.934]<br>t = 55.643<br>p < 0.001	r (570) = 0.580<br>[0.510, 0.646]<br>t = 17.006<br>p < 0.001

Table S2. *Two-tailed Pearson correlation of Bayes cue-integration, Situation-only and Face-only model across the four studies with bootstrapped 95% confidence intervals reported in the bracket.*

Study	Bayes cue-integration model	Situation-only model	Face-only model
Study 1	r (7850) = 0.840<br>[0.830, 0.849]<br>t = 137.15<br>p < 0.001	r (7850) = 0.865<br>[0.857, 0.873]<br>t = 153.04<br>p < 0.001	r (7850) = 0.660<br>[0.644, 0.677]<br>t = 77.942<br>p < 0.001
Study 2	r (570) = 0.778<br>[0.744, 0.809]<br>t = 29.581<br>p < 0.001	r (570) = 0.897<br>[0.879, 0.912]<br>t = 48.343<br>p < 0.001	r (570) = 0.559<br>[0.499, 0.612]<br>t = 16.077<br>p < 0.001
Study 3	r (570) = 0.798<br>[0.766, 0.828]<br>t = 31.614<br>p < 0.001	r (570) = 0.912<br>[0.894, 0.928]<br>t = 53.171<br>p < 0.001	r (570) = 0.559<br>[0.481, 0.629]<br>t = 16.038<br>p < 0.001
Study 4	r (570) = 0.784<br>[0.750, 0.816]<br>t = 30.119<br>p < 0.001	r (570) = 0.927<br>[0.911, 0.940]<br>t = 58.944<br>p < 0.001	r (570) = 0.534<br>[0.457, 0.606]<br>t = 15.081<br>p < 0.001

Table S3. *Bootstrapped 95% confidence intervals (CI) for pairwise difference in model correlations for each emotion category. CIs that do not include 0 suggest a significant difference between the two models and is denoted by the asterisk* (*).

Emotion	95% Confidence Interval for difference of correlation		
	Face-only - Sit-only model	Sit-only - Bayes cue-integration model	Bayes cue-integration - Face-only model
Amusement	[-0.088, 0]	[-0.083, -0.015] *	[0.061, 0.125] *
Anger	[-0.119, -0.032] *	[-0.056, 0.002]	[0.078, 0.129] *
Awe	[-0.559, -0.368] *	[0.053, 0.188] *	[0.253, 0.441] *
Contempt	[-0.28, -0.155] *	[-0.025, 0.055]	[0.158, 0.248] *
Disgust	[-0.289, -0.173] *	[-0.02, 0.053]	[0.174, 0.255] *
Embarrassment	[-0.698, -0.509] *	[0.114, 0.217] *	[0.371, 0.511] *
Fear	[-0.38, -0.239] *	[0.012, 0.08] *	[0.213, 0.316] *
Happiness	[-0.11, -0.027] *	[-0.065, -0.017] *	[0.078, 0.141] *
Interest	[-0.52, -0.351] *	[0.055, 0.16] *	[0.27, 0.389] *
Pride	[-0.479, -0.326] *	[0.071, 0.171] *	[0.226, 0.341] *
Sadness	[-0.192, -0.075] *	[-0.059, 0.007]	[0.12, 0.198] *
Shame	[-0.543, -0.369] *	[0.054, 0.147] *	[0.297, 0.422] *
Surprise	[-0.235, -0.112] *	[-0.03, 0.041]	[0.126, 0.21] *

Table S4. *Emotion categories ranked in descending order of most frequently experienced expressions in everyday life, and model correlation values for Bayes cue-integration, Face-only and Situation-only model, respectively.*

Ranks for Emotions			
Somerville & Whalen (2006)	Bayes cue-integration model	Face-only model	Situation-only model
**Happy**	**Happiness**	**Happiness**	**Fear**
Neutral	**Sadness**	Amusement	Pride
**Sad**	Amusement	**Anger**	Embarrassment
**Anger**	**Anger**	**Sadness**	**Sadness**
**Surprise**	**Fear**	**Surprise**	**Happiness**
**Disgust**	Contempt	Contempt	Shame
**Fear**	**Disgust**	**Disgust**	**Anger**
NA	**Surprise**	**Fear**	Awe
NA	Pride	Pride	Contempt
NA	Shame	Shame	**Disgust**
NA	Interest	Interest	Amusement
NA	Awe	Awe	Interest
NA	Embarrassment	Embarrassment	**Surprise**

Note: The range of emotion categories studied in prior work[1] is not completely comparable to the emotion categories in current work so we rank the 6 common emotion categories (bolded in the table) in descending order to perform Kendall's rank order correlation test (Table S5).

Table S5. *Results for two-tailed Kendall's rank order correlation to test for difference in rank orders for the mutual emotion categories (happy, sad, anger, fear, disgust, surprise) from Table S3. τ varies from +1 (ranking of two variables is similar) to -1 (ranking of two variables is in reverse order).*

	Rank orders for Bayes cue-integration correlations	Rank orders for Face-only correlations	Rank orders for Situation-only correlations
Ranks orders from Somerville & Whalen	$\tau = 0.600$, $T = 12$, $p = 0.136$	$\tau = 0.867$, $T = 14$, $p = 0.017$	$\tau = 0.067$, $T = 8$, $p = 1.00$

Note: There is a significant correlation between the rank order of emotions based on frequency of encountering expression[1] and the Face-only model ($\tau = 0.867$, $T = 14$, $p = 0.017$). This suggests that when inferring emotions people's reliance on facial cues tracks with the frequency of encountering expressions of those emotions in everyday life. People rely more on facial cues for emotion categories that are also more frequently encountered expressions in everyday life.

Age-related effects

Table S6. *Results for two-tailed Pearson correlation of cue-reliance and cue-integration with participants' age across studies 2-4.*

	Face-only model	Situation-only model	Bayes cue-integration model
Study 2	r (125) = -0.069 [-0.241, 0.106]<br>t = -0.776<br>p = 0.439	r (125) = 0.332 [0.167, 0.479]<br>t = 3.935<br>p < 0.001	r (125) = 0.056 [-0.119, 0.228]<br>t = 0.627<br>p = 0.532
Study 3	r (145) = -0.0003 [-0.162, 0.161]<br>t = -0.005<br>p = 0. 996	r (145) = 0.167 [0.005, 0.320]<br>t = 2.041<br>p = 0.043	r (145) = 0.054 [-0.109, 0.214]<br>t = 0.654<br>p = 0.514
Study 4	r (265) = 0.111 [-0.009, 0.228]<br>t = 1.826<br>p = 0.069	r (265) = 0.264 [0.149, 0.373]<br>t = 4.463<br>p < 0.001	r (265) = 0.094 [-0.026, 0.212]<br>t = 1.539<br>p = 0.125

Note: Given previous studies demonstrating age-related effects on how context influences processing of facial expressions[2], we also conducted an exploratory analysis to examine such effects. Results indicate that there was no statistically significant association of participants' age with their reliance on facial cues or integration of facial and situational cues across studies 2-4 (see Table S6). However, there was a small but significantly positive correlation between age and reliance on situational cues. This suggests that as people grow older, they are more likely to rely on situational information, consistent with previous findings[2]. Given the small but positive relationship between age and situation-reliance, we re-examined the association of situation-reliance with STEU-B controlling for age. Consistent with our initial results, we find positive association between situation reliance and people's situated understanding of emotions after controlling for age (Study 3: r (145) = 0.288, t = 3.614, p < 0.001; Study 4: r (265) = 0.312, t = 5.341, p < 0.001). This suggests there is a robust association between people's tendency to use situational information to infer other's emotions and their ability to understand the different emotions that are experienced across various social situations.

Table S7. *Test-retest reliability for cue-reliance (face-reliance and situation-reliance) and cue-integration estimates with 95% confidence intervals reported in the bracket. These estimates were computed using two-tailed tests of intra-class correlations after removing multivariate outliers detected using the Mahalanobis distance method.*

	Face-reliance	Situation-reliance	Cue-integration
Study 5	*ICC* = 0.300 [-0.182, 0.599]<br>F (106, 106) = 2.247<br>$p < 0.001$	*ICC* = 0.588 [0.125, 0.780]<br>F (106, 106) = 3.302<br>$p < 0.001$	*ICC* = 0.708 [0.495, 0.821]<br>F (106, 106) = 3.996<br>$p < 0.001$

Note: The number of outliers detected for face-reliance, situation-reliance, and cue-integration model estimates were three, three, and three respectively.

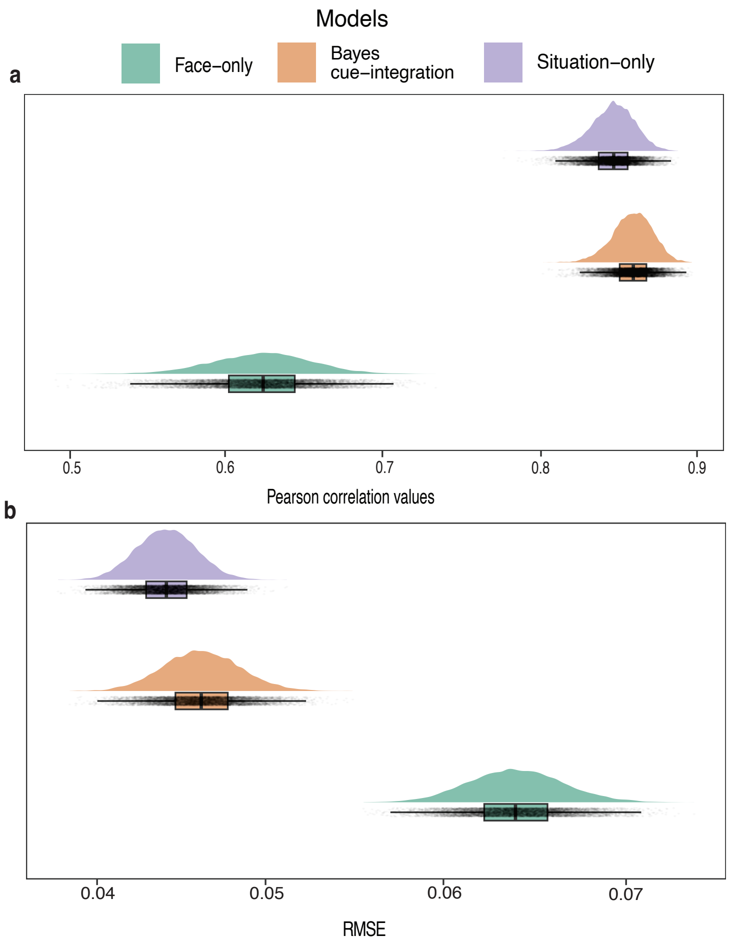

Figure S4 | Overall comparison of the three models using direct certainty ratings for a subset of stimuli (*n* = 44). a, Distribution of bootstrapped overall two-tailed Pearson model correlation values computed for each model derived from $n = 572$ pairs of observations – face-only (r (570) = 0.625, $t = 19.09$, $p < 0.001$, 95% CI: 0.560, 0.686), Bayes cue-integration (r (570) = 0.866, t = 41.41, $p < 0.001$, 95% CI: 0.839, 0.890), and Situation-only (r (570) = 0.854, $t = 39.14$, $p < 0.001$, 95% CI: 0.823, 0.880). Higher correlation values suggest better model fit. The face-only model correlation is significantly different from Bayes cue-integration (t (570) = 16.52, $p < 0.001$) and Situation-only models (t (570) = 9.94, $p < 0.001$) based on a two-tailed test of difference between two correlations, but there is no statistically significant difference between Bayes cue-integration and Situation-only model (t (570) = 1.06, $p = 0.290$). **b,** Overall root-mean-squared-error (RMSE) estimate for each model derived from $n = 572$ pairs of observations - face-only (0.063, 95% CI: 0.058, 0.069), Bayes cue-integration (0.044, 95% CI: 0.040, 0.049), and Situation-only (0.042, 95% CI: 0.039, 0.046). Lower RMSE values suggest better model fit. The center line in the box plot represents the mean correlation and mean RMSE values for the bootstrapped sample. The upper and lower limits of each boxplot represents the upper (75th percentile) and lower (25th percentile) quartiles of correlation or RMSE values for each model and the whiskers extending from the box represent the 1.5x interquartile range.

Rating distribution for face-only and situation-only conditions

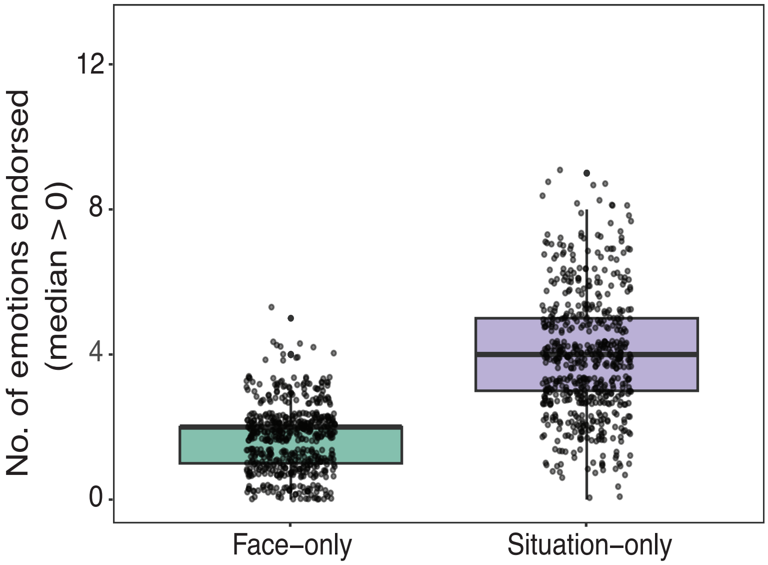

Figure S5 | Average emotions endorsed for face-only and situation-only conditions. Distribution of emotions endorsed for n = 604 stimulus in the face-only (M = 1.52, SD = 1.00) and situation-only (M = 3.89, SD = 1.68) condition. Median rating greater than zero for any emotion on a given stimulus is quantified as an endorsed emotion for that stimulus. The center line in the box plots represent the median number of emotions endorsed for each condition. The upper and lower limits of each boxplot represents the upper (75th percentile) and lower (25th percentile) quartiles of number of emotions endorsed. The whiskers extending from the box represent the 1.5x interquartile range while the black points are outliers for the given distribution.

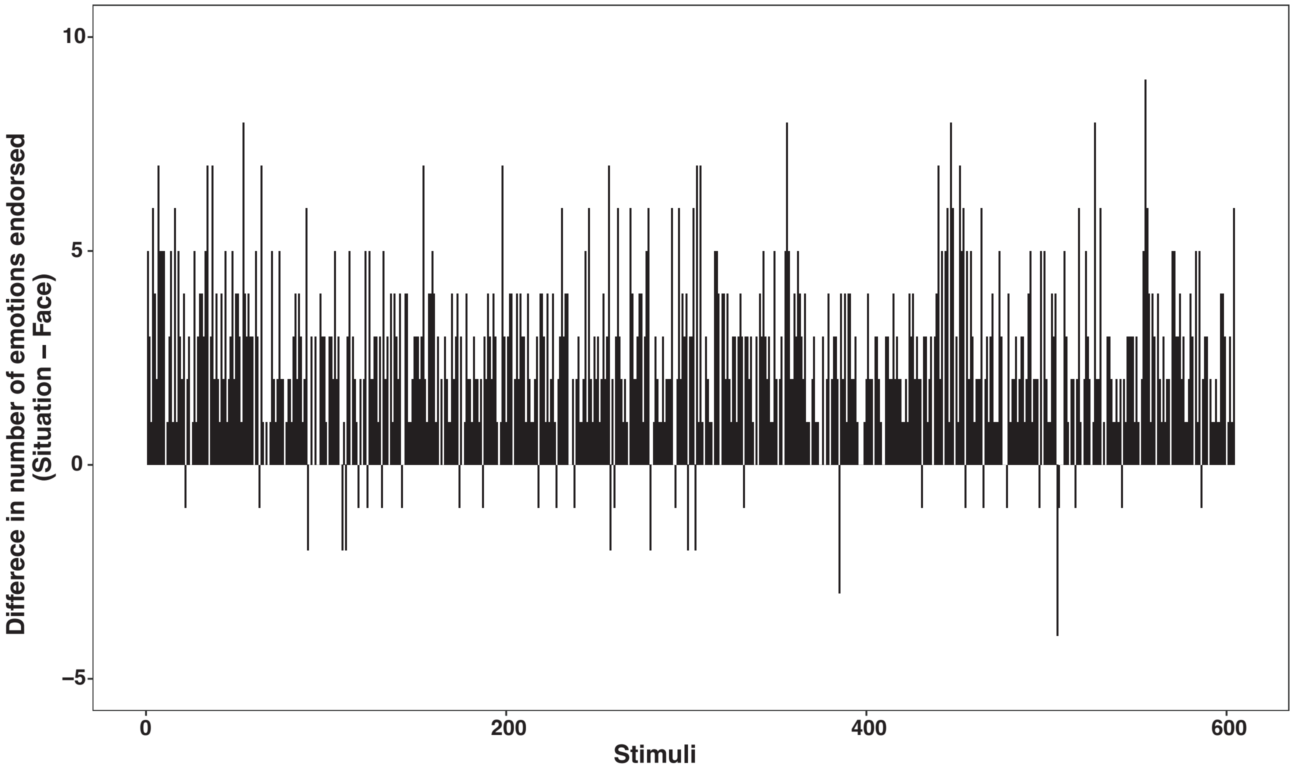

Figure S6 | Difference in number of emotions endorsed by stimuli. Number of emotions endorsed for n = 604 stimulus in the face-only condition is subtracted from those endorsed in situation-only condition. Median rating greater than zero for any emotion on a given stimulus is quantified as an endorsed emotion for that stimulus. Values greater than 0 suggest a greater number of emotions were endorsed for that stimulus in the situation-only condition compared to the face-only condition.

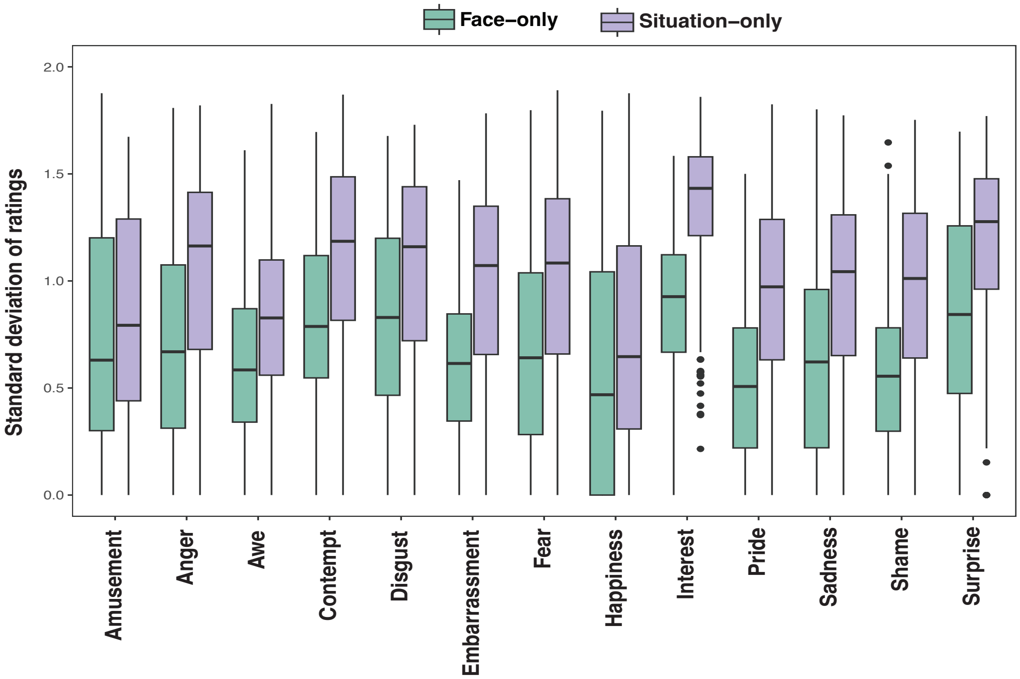

Figure S7 | Average variance in ratings for each emotion across the face-only and situation-only conditions. Distribution of variance in ratings for each emotion category $n = 604$ stimulus in the face-only and situation-only condition. The center line in the box plots represent the median variance for each emotion category and condition. The upper and lower limits of each boxplot represents the upper (75th percentile) and lower (25th percentile) quartiles of variance values. The whiskers extending from the box represent the 1.5x interquartile range while the black points are outliers for the given distribution. For emotion category, on average there is greater variance of ratings in the situation-only condition compared to the face-only condition.

Table S8. *Qualitative comparison of the order of emotions based on frequency of experience in everyday life*[1] *to the frequency of emotions perceived in everyday life (derived from 'prior data' collected in this manuscript) for the comparable list of emotions.*

Descending order of Emotion Experience Ranks: Sommerville, & Whalen (2006)	Descending order of Emotion Perception ratings from *Priors data*.
Happy	Happiness
Sad	Anger
Anger	Sadness
Surprise	Surprise
Disgust	Disgust
Fear	Fear

Table S9. *Demographic details for participants from Prior Data.*

Demographic Variable		Percentage of Participants Prior Data (N = 45)
Age		Mean = 38<br>SD = 13.88
Gender	Male	44.44
	Female	55.56
	Non-Binary	0
Educational Attainment	Less than a high school diploma	4.44
	High School Degree or Equivalent	22.22
	Some college	26.67
	Associate Degree	4.44
	Bachelor's Degree	26.67
	Postgraduate Degree	15.56
Race and/or Ethnicity	American Indian or Alaskan Native	0
	Asian	11.11
	Black or African American	2.22
	Native Hawaiian or Other Pacific Islander	0
	White	80
	Hispanic	0
	Latinx	0
	Other	6.67

Table S10. *Demographic details for participants from Studies 2-5 and archival data*[3].

Percentage of Participants in each sample	Age	Gender	Race and/or Ethnicity	Educational Attainment
Study 2 (*N* = 142)	Mean = 33.84 SD = 11.69	M = 47.18 F = 50.70 Non-Binary = 2.11	American Indian or Alaskan Native = 0 Asian = 10.56 Black or African American = 7.75 Native Hawaiian or Other Pacific Islander = 0.7 White = 76.06 Hispanic = 0 Latinx = 0 Other = 4.93	Less than a high school diploma = 1.41 High School Degree or Equivalent = 18.31 Some college = 23.94 Associate Degree = 7.75 Bachelor's Degree = 38.03 Postgraduate Degree = 10.56
Study 3 (*N* = 162)	Mean = 36.04 SD = 12.87	M = 34.57 F = 59.88 Non-Binary = 5.56	American Indian or Alaskan Native = 0 Asian = 5.56 Black or African American = 12.35 Native Hawaiian or Other Pacific Islander = 0.62 White = 71.6 Hispanic = 2.47 Latinx = 0 Other = 7.41	Less than a high school diploma = 0 High School Degree or Equivalent = 12.35 Some college = 25.93 Associate Degree = 12.35 Bachelor's Degree = 39.51 Postgraduate Degree = 9.88
Study 4 (*N* = 294)	Mean = 45.13 SD = 16.31	M = 47.96 F = 51.36 Non-Binary = 0.34	American Indian or Alaskan Native = 0 Asian = 5.1 Black or African American = 12.24 Native Hawaiian or Other Pacific Islander = 0.62 White = 71.43 Hispanic = 3.74 Latinx = 0 Other = 7.51	Less than a high school diploma = 0.34 High School Degree or Equivalent = 11.56 Some college = 23.13 Associate Degree = 11.56 Bachelor's Degree = 36.39 Postgraduate Degree = 16.33
Study 5 (*N* = 119)	Mean = 36.78 SD = 11.29	M = 47.06 F = 48.74 Non-Binary = 4.2	American Indian or Alaskan Native = 0 Asian = 5.88 Black or African American = 8.4	Less than a high school diploma = 0.84 High School Degree or Equivalent = 16.81 Some college = 26.05

			Native Hawaiian or Other Pacific Islander = 0<br>White = 65.55<br>Hispanic = 3.36<br>Latinx = 0<br>Other = 16.81	Associate Degree = 13.45<br>Bachelor's Degree = 31.09<br>Postgraduate Degree = 11.76
Archival Situation-only (*N* = 839)	Median = 35	M = 43.27<br>F = 56.38<br>Other = 3.58	White = 80.45<br>Black or African American = 10.25<br>Asian = 4.65<br>Other = 4.65	NA
Archival Face-only (*N* = 842)	Median = 35	M = 41.92<br>F = 57.36<br>Other = 7.13	White = 79.10<br>Black or African American = 9.14<br>Asian = 6.53<br>Other = 5.23	NA
Archival Joint-cue (*N* = 845)	Median = 35	M = 42.6<br>F = 56.8<br>Other = 5.92	White = 79.17<br>Black or African American = 7.93<br>Asian = 7.69<br>Other = 5.21	NA

Note: The sampling criteria across the archival data and empirical data collected in this project are highly similar. There is information on age, gender, race and ethnicity across the samples and the age of enrollment for all studies was restricted to 18-60 years. In the samples collected for this project, we ask an additional demographic question about educational attainment and have greater diversity in response options presented for race. Nevertheless, the distribution of sample across gender and race are largely comparable as observed from the percentage of sample reported in each category.

REFERENCES

1 Somerville, L. H. & Whalen, P. J. Prior experience as a stimulus category confound: an example using facial expressions of emotion. *Social cognitive and affective neuroscience* **1**, 271-274 (2006).

2 Ngo, N. & Isaacowitz, D. M. Use of context in emotion perception: The role of top-down control, cue type, and perceiver's age. *Emotion* **15**, 292 (2015).

3 Le Mau, T. *et al.* Professional actors demonstrate variability, not stereotypical expressions, when portraying emotional states in photographs. *Nature communications* **12**, 1-13 (2021).
