# Supplementary material for: Face and context integration in emotion inference is limited and variable across categories and individuals
Source: Nat Commun. 2024 Mar 19;15:2443. doi: 10.1038/s41467-024-46670-5 (PMC10948792; doi:10.1038/s41467-024-46670-5)
Supplement: Supplementary file 3 — Description of Additional Supplementary Files [file 41467_2024_46670_MOESM3_ESM.pdf]

Description of Additional Supplementary Files

File Name: Supplementary Data 1
Description: This data file contains the mean rating for each emotion category and stimulus from the Situation-only condition.
