# Supplementary material for: Face and context integration in emotion inference is limited and variable across categories and individuals
Source: Nat Commun. 2024 Mar 19;15:2443. doi: 10.1038/s41467-024-46670-5 (PMC10948792; doi:10.1038/s41467-024-46670-5)
Supplement: Supplementary file 5 — Reporting Summary [file 41467_2024_46670_MOESM5_ESM.pdf]

nature portfolio

Corresponding author(s): Srishti Goel, Maria Gendron

Last updated by author(s): Jan 30, 2024



# Reporting Summary

Nature Portfolio wishes to improve the reproducibility of the work that we publish. This form provides structure for consistency and transparency in reporting. For further information on Nature Portfolio policies, see our Editorial Policies and the Editorial Policy Checklist.

## Statistics

For all statistical analyses, confirm that the following items are present in the figure legend, table legend, main text, or Methods section.

| n/a | Confirmed | |
|---|---|---|
| ☐ | ☒ | The exact sample size (*n*) for each experimental group/condition, given as a discrete number and unit of measurement |
| ☐ | ☒ | A statement on whether measurements were taken from distinct samples or whether the same sample was measured repeatedly |
| ☐ | ☒ | The statistical test(s) used AND whether they are one- or two-sided<br>*Only common tests should be described solely by name; describe more complex techniques in the Methods section.* |
| ☐ | ☒ | A description of all covariates tested |
| ☐ | ☒ | A description of any assumptions or corrections, such as tests of normality and adjustment for multiple comparisons |
| ☐ | ☒ | A full description of the statistical parameters including central tendency (e.g. means) or other basic estimates (e.g. regression coefficient) AND variation (e.g. standard deviation) or associated estimates of uncertainty (e.g. confidence intervals) |
| ☐ | ☒ | For null hypothesis testing, the test statistic (e.g. *F*, *t*, *r*) with confidence intervals, effect sizes, degrees of freedom and *P* value noted<br>*Give P values as exact values whenever suitable.* |
| ☐ | ☒ | For Bayesian analysis, information on the choice of priors and Markov chain Monte Carlo settings |
| ☒ | ☐ | For hierarchical and complex designs, identification of the appropriate level for tests and full reporting of outcomes |
| ☐ | ☒ | Estimates of effect sizes (e.g. Cohen's *d*, Pearson's *r*), indicating how they were calculated |

*Our web collection on statistics for biologists contains articles on many of the points above.*

## Software and code

Policy information about availability of computer code

| | |
|---|---|
| Data collection | Data for this study was collected via an online survey platform called Qualtrics. |
| Data analysis | The analysis for all data was conducted using open source software R (version 4.3.0) and RStudio (2022.07.2 Build 576) . All data, analysis scripts and packages relied upon are available in OSF repository (https://osf.io/7e6j5/) along with the necessary code to downloaded all packages. Packages used across all analysis are: "ggplot2", "grid", "scales", "reshape2", "tidyverse", "corrgram", "pwr", "psych" ,"corrgram", "irr", "corrplot", "DescTools", "ppcor", "Routliers", "boot", "ggExtra", "ggpubr", "svglite", "ggdist", "ggridges", "RColorBrewer", "stats". |

For manuscripts utilizing custom algorithms or software that are central to the research but not yet described in published literature, software must be made available to editors and reviewers. We strongly encourage code deposition in a community repository (e.g. GitHub). See the Nature Portfolio guidelines for submitting code & software for further information.

## Data

Policy information about availability of data

All manuscripts must include a data availability statement. This statement should provide the following information, where applicable:

- Accession codes, unique identifiers, or web links for publicly available datasets
- A description of any restrictions on data availability
- For clinical datasets or third party data, please ensure that the statement adheres to our policy

All data (deidentified) collected and analyzed for studies 1-5 and supplementary materials are provided in the OSF repository: https://osf.io/7e6j5/. The archival

data (Le Mau et al., 2021) used in this manuscript has been made publicly available by the original authors on OSF (https://osf.io/m32ha/). We have included instructions in the readme file of our OSF repository on how to access that data and incorporate it into our analyses.

## Research involving human participants, their data, or biological material

Policy information about studies with human participants or human data. See also policy information about sex, gender (identity/presentation), and sexual orientation and race, ethnicity and racism.

| | |
|---|---|
| Reporting on sex and gender | Our analyses are not constrained to one sex or gender and sex or gender was not considered as variables of interest in this research. The research samples were recruited from Prolific using the 'balanced' sampling feature that evenly recruits participants from the male and female sex. The final samples are not always evenly split as data is removed for some people who fail attention checks, give low effort responses or are identified as outliers. In the demographics section of each study, participants were asked about their sex/gender identity (male, female, non-binary) and data on this is provided in the source data and summarized in the methods and supplementary materials. |
| Reporting on race, ethnicity, or other socially relevant groupings | Our analyses are not constrained to a particular social group. The samples were recruited online using the Prolific platform. We collected self-reports for the following demographic variables - Race/Ethnicicty (American Indian or Alaskan Native, Asian, Black or African American, Native Hawaiian or Pacific Islander, White, Hispanic, Latinx, Other/Mixed Race) and educational qualification (less than a high school diploma, high school degree or equivalent, some college, associate degree, bacherlor's degree, postgraduate degree). Data on these variables are provided for each sample and summarized in the methods and supplementary materials. |
| Population characteristics | Research samples for studies 1, 2, 3 and 5 were recruited using convenience sampling strategy online and consisted of healthy adults between the age of 18 - 60 years who are native English speakers and residing in the United States. The samples were also approximately evenly distributed across sex. We set such criteria to ensure consistency in participants' cultural and linguistic background, as different backgrounds may impact emotion inferences, the focus of the study. Sample for study 4 was recruited using the 'representative' sampling feature on Prolific that recruits a representative sample for age (18 - 150), sex (male, female) and ethnicity (asian, black, mixed, other, white). |
| Recruitment | Participants were recruited using an Online recruitment platform called Prolific. This platform recruits a relatively more diverse sample compared to college students especially along educational qualification and age but there likely is self-selection bias as only people with electronic devices and access to Internet are able to access this platform and participate in these studies. |
| Ethics oversight | All studies comply with the ethical guidelines for conducting human subjects research and were administered under the exempt protocol approved by the Yale University Institutional Review Board (IRB #: 2000028669). Participants across all studies provided informed consent before participating in the study and were compensated a prorated amount for the duration of task using the then Prolific suggested hourly rate marked as 'Good' ($10-12/hr). |

Note that full information on the approval of the study protocol must also be provided in the manuscript.

# Field-specific reporting

Please select the one below that is the best fit for your research. If you are not sure, read the appropriate sections before making your selection.

☐ Life sciences ☒ Behavioural & social sciences ☐ Ecological, evolutionary & environmental sciences

For a reference copy of the document with all sections, see nature.com/documents/nr-reporting-summary-flat.pdf

# Behavioural & social sciences study design

All studies must disclose on these points even when the disclosure is negative.

| | |
|---|---|
| Study description | All five studies are behavioral studies with quantitative data that includes ratings on a likert-type or rating scale. |
| Research sample | Study 1 archival data (Le Mau et al., 2021, total N = 2526) included participants who provided ratings for one in 3 different conditions – face-only (N=842, median age = 35, 41.92% male, 57.36% female, 79.1% White, 9.14% Black, 6.53% Asian, 5.23% other), situation-only (N=839, median age = 35, 43.27% male, 56.38% female, 80.45% White, 10.25% Black, 4.65% Asian, 4.65% other), face and situation combined (N=845, median age = 35, 42.6% male, 56.8% female, 79.17% White, 7.93% Black, 7.69% Asian, 5.21% other)<br><br>For study 1 priors data we recruited 45 native English-speaking participants recruited from the US (mean age = 38-year, SD age = 13.88, 44.44% male, 55.56% female, 80% white, 2.22% Black or African American, 11.11% Asian).<br><br>In study 2 we recruited 150 native English-speaking participants between the age of 18-60 years from the US. Eight people did not complete the study, leaving a total sample of 142 participants (Mean age = 33.84, SD age = 11.69; 50.7% female, 47.18% male and 2.11% non-binary; 76.05% White, 10.56% Asian, 7.75% Black or African American).<br><br>In study 3 we recruited 168 native English-speaking participants between the age of 18-60 years from the US. Six people failed attention checks built in the task, so their data was removed from any analysis, leaving a sample of 162 individuals (Mean age = 36.04, SD age = 12.87; 59.88% female, 34.57% male and 5.56% non-binary; 71.6% White, 12.35% Black or African American, 5.56% Asian) |

In study 4 we recruited a nationally representative sample of 303 participants from the US. Nine people failed attention checks built in the task, so their data was removed from any analysis, leaving a sample of 294 individuals (Mean age = 45.13, SD age = 16.31; 51.36% female, 47.96% male and 0.34% non-binary; 71.43% White, 12.24% Black or African American, 5.1% Asian)

In study 5 we recruited 136 native English-speaking participants between the age of 18-60 years from the US. Seventeen people dropped out as they did not complete Session 2 so their data was removed from any analysis, leaving a sample of 119 individuals (Mean age = 36.78, SD age = 11.29; 48.74% female, 47.06% male and 4.20% non-binary; 65.55% White, 8.40% Black or African American, 5.88% Asian)

Sampling strategy

For study 1 priors data we recruited 45 participants to approximately match the archival dataset where on average ~40 participants responded to a given stimulus.

Sample size for study 2 was determined a priori using power analysis for bivariate correlations controlling for multiple comparisons using G*Power. The results indicated that with a sample of 149 we have enough power to detect a small-medium effect size (r = 0.3). We therefore recruited 150 participants for this study.

Sample size for study 3 was also predetermined using power analysis for bivariate correlations controlling for multiple comparisons using G*Power. The results indicated that with a sample of 139 we have enough power to detect a small-medium effect size (r = 0.25). We oversampled for an additional 20% of data to account for exclusions and attrition based on previous study, resulting in a total sample of 168 individuals.

Study 4 (pre-registered) sample size was planned to recruit a nationally representative sample from the US and provide a more stable estimate of previously observed effect sizes as correlations tend to stabilize at N = 260. We recruited more than 260 participants to meet the minimal sample size (N = 300) on Prolific to recruit a nationally representative sample. We also conducted an a priori power analysis for bivariate correlations to detect the effect size for the relationship between STEU and situation-reliance that was observed in the previous study (r = 0.3), controlling for multiple comparisons (alpha = 0.05/3) using G*power. The results indicated that with a sample size of 95 we have enough power to detect the expected effect size (r = 0.3). The planned sample size therefore met this sample size requirement while also estimating stabilized effect sizes.

Study 5 sample size was pre-determined using power analysis for bivariate correlations controlling for multiple comparisons using G*Power. The results indicated that with a sample of 102 we have enough power to detect a desirable effect size (ICC: r = 0.75). We oversampled for an additional 25% of data to account for dropouts, exclusions and attrition based on previous studies, resulting in a total sample of 136 individuals.

Data collection

Participants completed tasks without the presence of a researcher using their own computers as data was collected Online.

Timing

Study 1 priors data: Dec 5, 2020 - Jan 13, 2021
Study 2 data: March 4, 2021 - March 11, 2021
Study 3 data: May 18, 2022 - May 26, 2022
Study 4 data: June 16, 2022 - June 28, 2022
Study 5 data: Dec 13, 2022 - Jan 11, 2023

Data exclusions

In study 1, we removed participants with spurious data i.e., absence of ratings or identical ratings for more than 80% of data, from the archival (N = 13) and priors task data (N = 1) leaving a total of 2513 and 44 participants in the respective datasets for analysis. For studies 2 - 5, we removed spurious data from participants who provided accidental, incorrect, or had missing ratings for more than 10% of their data. In study 2, 8 participants did not complete the study and spurious data from 11 participants was removed leaving a sample size of 131 participants. Further, 2 subjects were identified as outliers for multivariate outlier detection using the Mahalanobis distance and their data was removed resulting in 129 participants for final analysis. In study 3, spurious data from 10 participants was removed leaving a sample size of 152 participants. Further, 4 subjects were identified as outliers for multivariate outlier detection using the Mahalanobis distance and their data was removed resulting in 148 participants for final analysis. In study 4, spurious data from 19 participants was removed leaving a sample size of 275 participants. Further, 7 subjects were identified as outliers for multivariate outlier detection using the Mahalanobis distance and their data was removed resulting in 268 participants for final analysis. Study 2 required participants to complete two sessions, two weeks apart. We recruited participants (N = 136) to account for some attrition. Seventeen people dropped out as they did not complete Session 2 so their data was removed from any analysis, leaving a sample of 119 individuals. Spurious data from 9 participants was also removed resulting in 110 participants for final analysis.

Non-participation

No participants declined participation across the studies. 8 participants did not finish study 2 after consenting so their data was removed. 17 people consented for study 5 but did not return and complete the session 2 so their data from first time point was removed and they were not included in the analysis.

Randomization

There were no experimental groups and therefore no randomization of participants in the collected data. All participants completed all tasks in each study session. The order for different tasks and scales or trials within tasks was randomized within a study session for each participant.

# Reporting for specific materials, systems and methods

We require information from authors about some types of materials, experimental systems and methods used in many studies. Here, indicate whether each material, system or method listed is relevant to your study. If you are not sure if a list item applies to your research, read the appropriate section before selecting a response.

## Materials & experimental systems

| n/a | Involved in the study |
| --- | --- |
| ☒ | ☐ Antibodies |
| ☒ | ☐ Eukaryotic cell lines |
| ☒ | ☐ Palaeontology and archaeology |
| ☒ | ☐ Animals and other organisms |
| ☒ | ☐ Clinical data |
| ☒ | ☐ Dual use research of concern |
| ☒ | ☐ Plants |

## Methods

| n/a | Involved in the study |
| --- | --- |
| ☒ | ☐ ChIP-seq |
| ☒ | ☐ Flow cytometry |
| ☒ | ☐ MRI-based neuroimaging |
